# Protective Role of *IL7A*-197 A/G Heterozygosity in the Development and Severity of Colorectal Cancer in the Bulgarian Population

**DOI:** 10.3390/medicina58111632

**Published:** 2022-11-11

**Authors:** Elina Aleksandrova, Tatyana Vlaykova, Julian Ananiev, Maya Gulubova

**Affiliations:** 1 Department of General and Clinical Pathology, Forensic Medicine and Deontology, Medical Faculty, Trakia University, 6000 Stara Zagora, Bulgaria; 2 Department of Chemistry and Biochemistry, Medical Faculty, Trakia University-Stara Zagora, 6000 Stara Zagora, Bulgaria

**Keywords:** IL-17A, IL-17F, polymorphism, genotype, colorectal cancer, disease susceptibility

## Abstract

*Background*: Interleukin (IL)-17A and IL-17F, expressed mainly by a novel subset of CD-positive (+) T-helper (Th) cells of the immune system, has been closely related to inflammatory conditions underlying colorectal cancer pathogenesis. Accordingly, we conducted a case-control study to investigate the association of common single nucleotide polymorphisms (SNP) in the *IL17A* and *IL17F* genes (rs2275913 and rs763780, respectively) with the susceptibility and severity of CRC patients from the Bulgarian population. *Methods and Materials*: 136 patients with histologically confirmed CRC diagnosis and 116 healthy individuals were recruited in the present study. Genotypes were determined by the restriction fragment length polymorphism-polymerase chain reaction (RFLP-PCR) technique. *Results*: The *IL17A* heterozygous A/G-genotype was overrepresented among the control group (*p* = 0.003). Additionally, the carriers of the heterozygous A/G-genotype had a 2.39-fold lower risk for CRC compared to the G/G-genotype (OR = 0.418, *p* = 0.006). Our results also indicated that in the advanced CRC stages (III + IV) the heterozygous genotype (A/G) appeared to be less frequent (*p* = 0.024, χ^2^-test). Among the patients with detected distant metastases, the A/G-carriers were the smallest part (14.3%) compared to the homozygous genotypes A/A (42.9%) and G/G (42.8%), *p* = 0.006. There was no association of the studied *IL17F* rs763780 SNP with susceptibility and severity of CRC among the studied subjects, although the heterozygous C/T-carriers had shorter median survival compared to the T/T-carriers (*p* = 0.129). *Conclusions*: Our study finds a protective role of heterozygosity for the *IL17A*-197A/G SNP and negative effects of the A-allele on CRC progression.

## 1. Introduction

Colorectal cancer (CRC) poses a significant challenge to the global public health systems as the disease is the third most common cancer and is the second leading cause of cancer-related deaths worldwide according to the GLOBOCAN 2020 statistics [1].

CRC is a heterogeneous disease in both biological and clinical terms. The sequence of neoplastic progression (classical polyp-adenoma-carcinoma pathway) is associated with the accumulation of mutations and other genetic and epigenetic changes in cells. Despite the complex etiology of CRC, extensive epidemiological observations provide evidence of the association between chronically inflamed tissue and its subsequent malignant transformation [2]. The mechanisms of the inflammatory process disrupt the organization of the stromal environment and lead to genomic instability [3]. Although its fundamental role in the body is protection, in order to repair damaged tissue the inflammatory immune response remodels the tumor microenvironment and leads to changes in cytokine profiles and concentrations, both locally and systemically.

Interleukins-17A and -17F (IL-17A, IL-17F) belong to a relatively recently described cytokine superfamily, including another four members IL-17B, IL-17C, IL-17D and IL-17E. Each of these polypeptides contains 163–202 amino acid residues and has a molecular weight of about 25–30 kDa. Their structure is characterized by the presence of four conservative cysteine residues, which most likely form intermolecular disulfide bridges [4]. IL-17A and IL-17F share the highest homology in structure and function compared to other members of the cytokine family [5]. Both molecules function through the IL-17RA receptor, a ubiquitous transmembrane protein that activates MAP-kinase and the NF-kB signaling pathways in cells. Following signal transduction, IL-17A and IL-17F induce the production of proinflammatory cytokines, chemokines and prostaglandins from the inflammatory cells of the immune system [6]. These are represented by the populations CD4+ and CD8+ T-lymphocytes, B-cells, macrophages and NK-cells, which through their cytokines can suppress or induce the growth of CRC. For example, IFN-γ secreted by Th-1 CD4+ and CD8+ cells activates cytotoxic immunity, inhibits tumor growth, and is associated with a reduced risk of relapse in CRC [7]. In contrast, cytokines produced by T helper-17 cells (Th-17), which are a subpopulation of CD4+-helper T-lymphocytes, demonstrate tumorigenic effects, and tumor infiltration with Th-17 cells adversely affecting the prognosis in CRC [8].

The expression of IL-17 is a characteristic feature of Th-17 cells. In healthy individuals, about 1% of the populations of CD4+ T cells in the peripheral blood are Th-17 cells [9]. Their number increases significantly in pathological changes, such as tumor formations, and this is associated with the production of large amounts of IL-17 [10]. The differentiation of Th-17 cells differs from that of Th1, Th2 and T-reg cells in the unique transcription factors involved in the process, as well as in the cytokine profile in the environment [11]. 

Depending on the experimental setting, Th-17 cytokines may have a positive or negative effect on the malignant potential of tumor cells [12]. The degree of infiltration of CRC with IL-17A-producing CD4+ lymphocytes correlates with a worse prognosis for disease outcome [13]. Th-17 cells co-expressing the transcription factors ROR-γt and FOXP3 and producing IL-17A are abundant in patients with CRC [14]. These cells have immunosuppressive effects that support tumor progression. In addition, many studies have demonstrated the overexpression (at mRNA and functional protein levels) of the cytokines IL-17A, IL-17F, IL-21 and IL-22 in the tumor nest in patients with CRC compared to nearby unaffected intestinal mucosa [15,16]. Moreover, significantly lower survival was observed in patients with CRC who were found to have increased infiltration into the IL-17A tumor [7].

Given the above-described key role of IL-17A and IL-17F in the pathogenesis of CRC, it would be reasonable to assume that increased expression of Th-17 cytokines in patients with CRC might be associated with genetic polymorphisms that determine individual predisposition to the disease. The *IL17A* and *IL17F* genes are located on the short arm of the human chromosome 6–6p12. Numerous functional polymorphisms along the two genes have been identified, two of which have been studied in multiple populations. A single nucleotide polymorphism (G197A SNP; rs2275913) has been identified in the 5′ region of the *IL17A* gene promoter, which affects gene expression [17]. A polymorphism (T7488C, rs763780) has been described in the coding region of the *IL17F* gene, which is a missense mutation that affects the structure of the coded polypeptide, and therefore, its function. Both polymorphisms have been reported to be associated with the risk of gastric cancer [18], breast cancer [19], ulcerative colitis [20], Crohn’s disease [21], thyroid [22] and CRC [23]. In the available scientific literature, these studies have been performed among groups of patients mainly from Asian populations. Therefore, the objective of the present study aimed to determine whether these polymorphisms affect the susceptibility, clinical outcome and survival of CRC in Caucasians from the Bulgarian population.

## 2. Materials and Methods

### 2.1. Study Subjects

A total of 136 patients with histologically confirmed CRC diagnosis were retrospectively studied. Patients were recruited from the University Hospital “Prof. Stoyan Kirkovich”, Stara Zagora, Bulgaria. The study protocol was approved by the Ethics committee at Medical Faculty, Trakia University, Stara Zagora, Bulgaria (Protocol No. 9/15.05.2019). Fresh biopsy tissue from the patients was collected during surgery in accordance with approved guidelines and regulations. Samples were flash-frozen and stored at −80 °C until used for DNA extraction.

The tumor stage was classified according to the American Joint Committee on Cancer (AJCC) guidelines (AJCC manual, 8th ed, 2017). All patients were unrelated Caucasian individuals and were all untreated prior to surgery.The cancer-free control group consisted of 116 healthy individuals from the general population of Stara Zagora, Bulgaria. Control subjects were selected with no history of cancer, or autoimmune/inflammatory diseases (including diabetes mellitus, rheumatoid arthritis, lupus erythematosus, or inflammatory bowel disease). Blood was collected in EDTA-treated tubes and was subsequently frozen and stored at −80 °C until DNA extraction was performed.

### 2.2. DNA Extraction

Genomic DNA was extracted from fresh frozen tissues of tumor biopsies using a genomic DNA purification kit (NucleoSpin Tissue, Macherey-Nagel, Duren, Germany) and DNA from the controls was isolated from blood samples (NucleoSpin Blood L, Macherey-Nagel, Duren, Germany) in accordance with the manufacturer’s instructions. Extracted DNA was stored at –80 °C until further use. The concentration of genomic DNA was measured spectrophotometrically at 260 nm by NanoVue Spectrophotometer (GE Healthcare, Little Chalfont, UK). The ratio of absorptions at 260 versus 280 nm was calculated to assess the purity of DNA samples.

### 2.3. Genotyping

Genotyping for the *IL17A*-197 A/G (rs2275913) and *IL-17F* 7488 T/C (rs763780) single nucleotide polymorphisms was performed by restriction length polymorphisms (RFLP)-PCR assay. Amplification of the region surrounding the polymorphic site for rs2275913 and rs763780 was achieved with the following forward and reverse primer pairs, respectively: 5′-AACAAGTAAGAATGAAAAGAGGA-3′ 5′-CCCCCAATGAGGTCATAGAAGAA-3′ and 5′-ACCAAGGCTGCTCTGTTTCT-3′ 5′-GGTAAGGAGTGGCATTTCCTA-3′, purchased from Thermo Fisher Scientific (Waltham, MA, USA). The PCR amplification was performed in a final volume of 25 µL mixture containing: approx. 100 ng DNA template, 1 µM of each primer, 100 µM of each dNTP, 2.5 mM MgCl_2_ and 1U Taq DNA polymerase and 10× Taq Buffer (Thermo Fisher Sci, USA). PCR conditions were as follows: denaturation at 94 °C for 3 min, followed by 35 cycles of denaturation at 94 °C for 1 min, annealing at 58 °C for rs2275913 and 54 °C for rs763780, extension at 72 °C for 30 s and final extension at 72 °C for 7 min. Restriction enzymes *XagI* (*EcoNI*) and *HinlII* (*NlaIII*) were purchased from Thermo Fisher Scientific (USA). PCR products were digested overnight with 10 U/reaction of restriction enzymes at 37 °C. Details on the restriction reactions are presented in Table 1.

PCR products and restriction fragments were visualized on 3% agarose gel stained with 0.5 mg/mL ethidium bromide (Acros Organics, New Jersey, USA)). A heterozygous control template and negative control were applied in each PCR run to ensure accuracy.

### 2.4. Statistical Analysis

Statistical analysis was performed on SPSS software v. 25 (IBM Corporation, Armonk, New York, USA). Continuous variables were presented as the mean, standard error of mean, median and ranges. They were assessed for normality of the distribution by Kolmogorov–Smirnov’s test and Shapiro–Wilk’s W-test. The 2 × 3 and 2 × 2 cross-tables were analyzed by Chi-square test (χ^2^ test) in order to determine the frequency of manifestation of the categorical variables. When needed, Fisher’s exact test was used. The correlations between the quantitative variables were evaluated by Pearson’s or Spearman’s test depending on the distribution (normal or non-normal, respectively). The Hardy–Weinberg equilibrium was tested among the controls with chi-squared tests.

Binary logistic regression analysis was applied for assessing the odds ratios and 95% confidence interval (CI) when analyzing the effect of the genotypes on the risk for the development of CRC. Cumulative survival curves were drawn by the Kaplan–Meier method and the difference between the curves was analyzed by the Mantel–Cox (log-rank) test. *p*-values less than 0.05 were considered statistically significant.

For the construction of haplotypes and determining haplotype frequencies, the PHASE v.2.1 software product was used.

## 3. Results

### 3.1. Baseline Characteristics of CRC Cases and Controls

The CRC patients’ group was composed of 67 (49.3%) male and 69 (50.7%) female individuals with a mean age of 67.51 (±8.06) and 68.71 (±8.73), respectively, without significant age differences (*p* = 0.420, *t*-test). The mean age of the total group was 68.14 years (±8.33). The demographic and available clinical data of the studied CRC cases are listed in Table 2.

The controls consisted of 59 (50.9%) male and 57 (49.1%) female individuals with mean ages of 59.8 (±7.9) years and 63.1 (±3.1) years, respectively. The mean age of the group was calculated as 61.38 years (±5.82). There were no significant age differences between the male controls versus females (*p* = 0.408, *t*-test).

### 3.2. -197 G/A SNP (rs2275913) in IL17A Gene Is Associated with CRC Risk and Severity of the Disease

**Figure 1:**
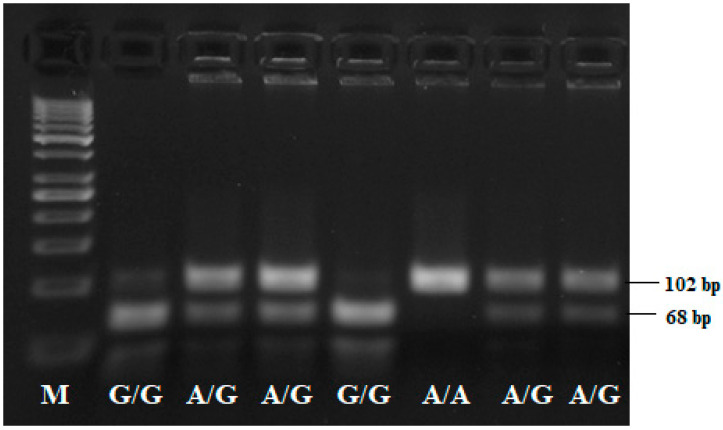
RFLP-PCR electropherogram representing the *IL17A*-197 A/G genotypes. A/A-genotype was detected with the uncut 102 bp DNA fragment; the A/G-genotype was visualized with two bands of 102 bp and 68 bp; the homozygous G/G-genotype exhibited a 68 bp band. The 38 bp fragment was too short to be detected. The M line was the 50 bp DNA marker.

Genotype and allelic frequencies of the -197 G/A SNP in the promoter of the *IL17A* gene were successfully determined in 104 CRC patients and 106 control individuals. After PCR was performed, the specific primer pair amplified a 102 bp DNA fragment containing a restriction site recognized by the *XagI* restriction enzyme. Allelic discrimination was performed on a horizontal agarose gel as follows: the A/A-genotype fragment remained uncut (102 bp); the G/A-genotype fragment yielded three fragments after restriction enzyme digestion—102 bp, 68 bp and 34 bp and the G/G-genotype fragment generated two fragments of 68 bp and 34 bp. Visualization of the DNA fragments via agarose gel electrophoresis after restriction enzyme digestion is presented in Figure 1. 

Genotype and allelic frequencies for the investigated SNP among CRC patients and healthy donors are described in Table 3. 

**Figure 2:**
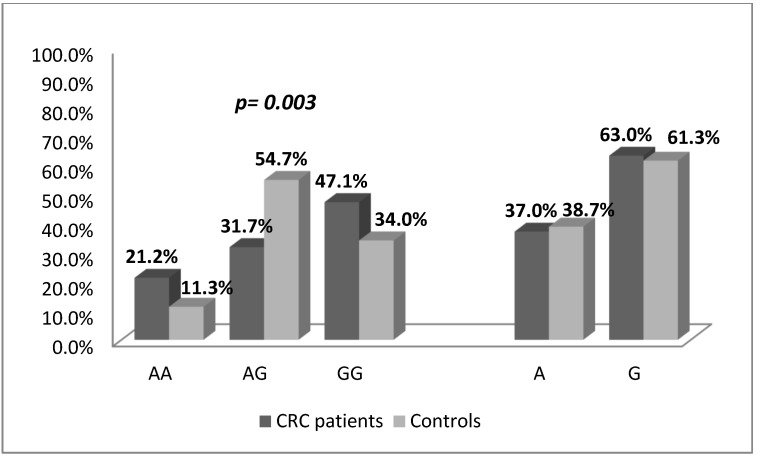
Distribution of genotype and allelic frequencies according to the *IL17A*-197 A/G SNP among CRC cases and controls. The heterozygous A/G-genotype was significantly more frequent among the control group (*p* = 0.003, χ^2^-test).

The distribution of genotypes among the controls was in agreement with the expected values fitting in the Hardy–Weinberg equilibrium (χ^2^ = 2.497, *p* = 0.114). Analysis of the genotype distribution in patients and controls revealed a higher frequency of the heterozygous A/G-genotype among the controls (*p* = 0.003, χ^2^ = 11.78), Figure 2. 

After logistic regression was performed, we found that the carriers of the heterozygous A/G-genotype had a 2.39-fold lower risk for CRC compared to the carriers of the G/G-genotype (OR = 0.418, *p* = 0.006). Additionally, the dominant model for genotype distribution (G/G vs A/G + A/A) showed that the carriers of the A-containing genotypes had a 1.73-fold lower CRC risk (OR = 0.577, *p* = 0.057), Table 2 and Table 4.

**Figure 3:**
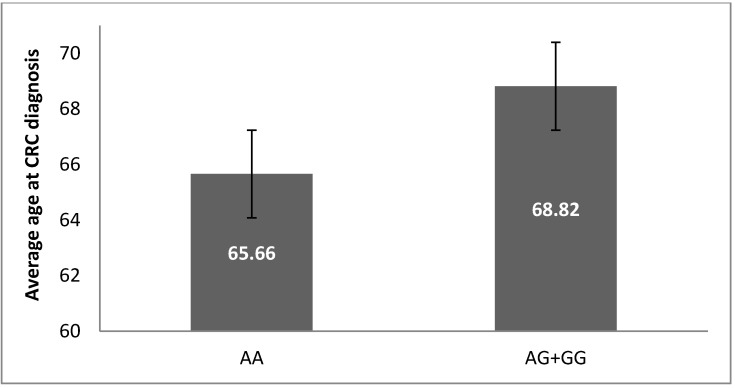
Association of *IL17A*-197 A/G genotypes and average age of diagnosis among CRC patients. A tendency for lower age at diagnosis was observed for the carriers of the homozygous A/A-genotype.

According to the age of diagnosis among CRC patients, we found a tendency for a lower age for the homozygous A/A-genotype carriers versus the A/G- and G/G-genotypes (*p* = 0.165, *t*-test), Figure 3.

**Figure 4:**
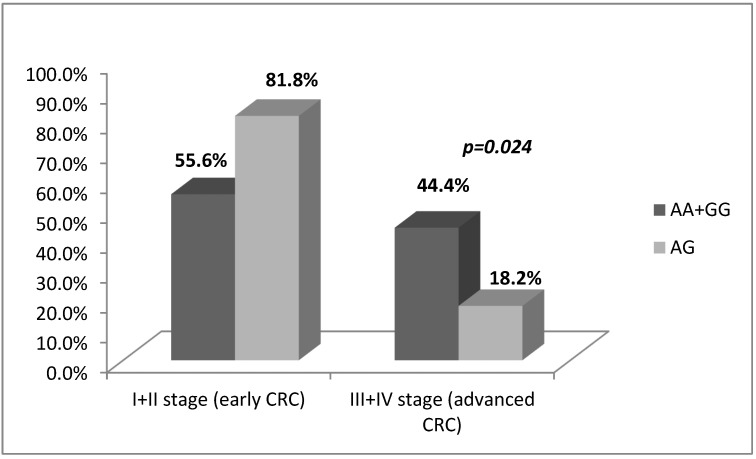
*IL17A*-197A/G genotype associations according to CRC stage. The heterozygous genotype was significantly less frequent in advanced CRC stage.

Furthermore, we investigated the association between the studied SNP and the severity of the disease. In the advanced CRC stages (III + IV) the heterozygous genotype (A/G) appeared to be less frequent compared to the homozygous genotypes (A/A + G/G), (OR = 0.278, *p* = 0.024, χ^2^-test), Figure 4.

**Figure 5:**
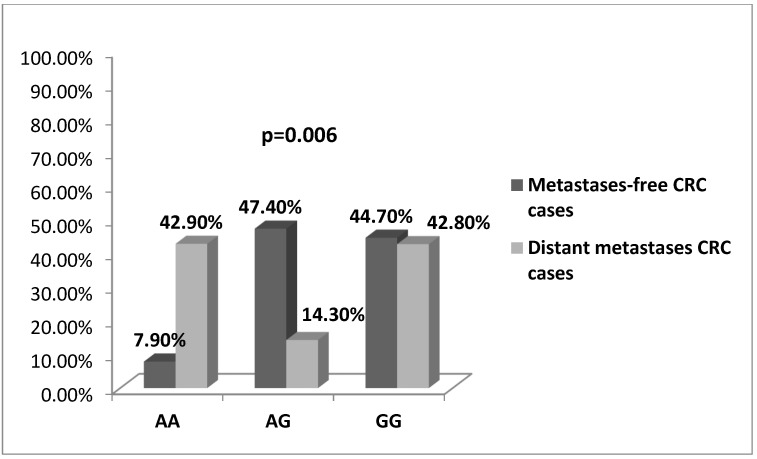
Association between metastatic status of CRC patients and genotypes for the *IL17A*-197 A/G polymorphism. In the group of metastases-free patients the A/A-genotype was less frequent and the heterozygous genotype was the smallest part among patients with detected distant metastases. (*p* = 0.006, χ^2^-test).

The genotype distribution of rs2275913 among CRC cases was also associated with the presence of distant metastases. It appeared that among the patients with detected distant metastases the A/G-carriers were the smallest part (14.3%) compared to the homozygous genotypes A/A (42.9%) and G/G (42.8%). It is noteworthy that the A/A-genotype was less frequent among the metastases-free patients 7.9% versus A/G- and G/G-genotypes (47.4% and 44.7%, respectively), (OR = 0.185, *p* = 0.006, χ^2^-test), Figure 5.

**Figure 6:**
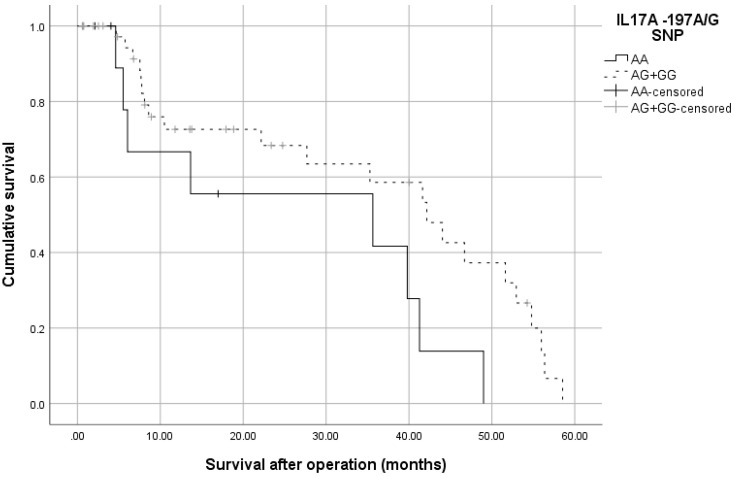
Kaplan–Meier survival curve of CRC patients according to *IL17A*-197A/G genotypes. The A/A-carriers had significantly shorter survival of 26.4 months compared to the A/G- and G/G-genotypes 35.9 months, (*p* = 0.039, Log-rank test).

The survival rates of the studied subjects were evaluated with respect to the *rs2275913* SNP. The carriers of the A/A-genotype had a shorter overall survival of 26.4 months compared to the G-allele containing genotypes 35.9 months (log-rank test, *p* = 0.039), Figure 6.

### 3.3. Distribution of 7488 T/C SNP (rs763780) in IL17F Gene

**Figure 7:**
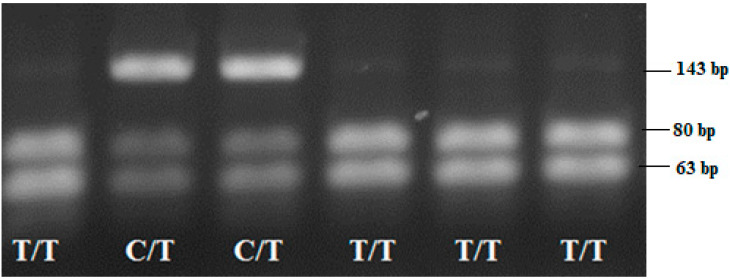
RFLP-PCR electropherogram representing the genotypes according to the *IL17F* 7488 T/C SNP: T/T-genotype was visualized with two DNA bands (80 bp and 63 bp); C/T-genotype exhibited three bands (143 bp, 80 bp and 63 bp) and the C/C-genotyped (143 bp) was not observed among the studied group.

According to the *rs763780* SNP, 113 CRC cases and 116 control individuals were successfully genotyped. The specific primers amplified a DNA fragment of 143 bp containing the 7488 T/C polymorphic sites in the *IL17F* gene. After RFLP analysis with the restriction enzyme *NlaIII*, the T/T-genotype was visualized on agarose gel with two bands, 80 bp and 63 bp, the T/C-genotype with three bands: 143 bp, 80 bp and 63 bp, and the C/C-genotyped exhibited with the undigested 143 bp fragment, Figure 7.

The distribution of genotype frequencies was not significantly different between CRC cases (T/T 94.7%, T/C 5.3%) and the control group (T/T 94.8%, T/C 5.2%) (*p* = 0.963, χ^2^-test). Data are presented in Table 5. The distribution of genotypes among the controls was in agreement with the expected values fitting in the Hardy–Weinberg equilibrium (χ^2^ = 0.082, *p* = 0.775).

We found no association of the studied polymorphism with selected clinical characteristics of the patients, such as CRC stage, presence of lymph node metastases, or distant metastases, Table 6.

**Figure 8:**
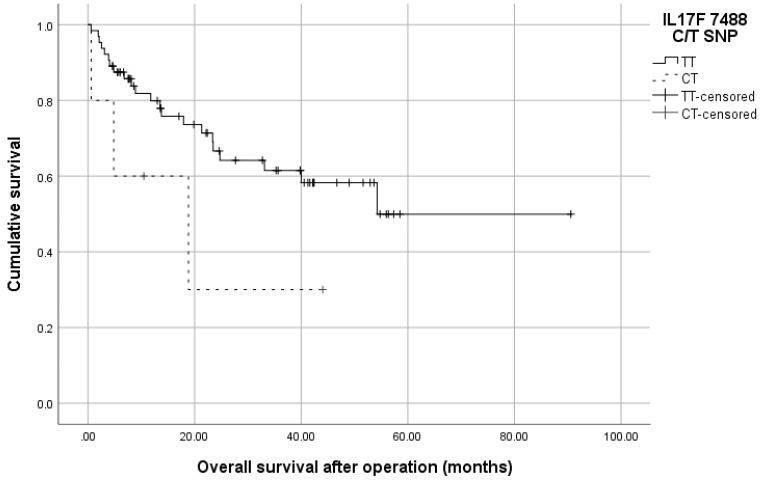
Kaplan–Meier survival curve of CRC patients according to *IL17F* 7488T/C genotypes. The C/T-carriers had shorter survival of 19.95 months compared to the T/T-genotype 56.12 months, (*p* = 0.039, Log-rank test).

Analysis of the survival rates of the patients showed a tendency for shorter overall survival of 19.95 months for the C/T-genotype carriers versus 56.12 months for the T/T-genotype (Log-rank test, *p* = 0.127), Figure 8.

### 3.4. Haplotypes Analyses

Due to the localization of the *IL17A* and *IL17F* genes on the same chromosome, haplotype analysis was performed for the studied polymorphisms. The frequencies of the constructed haplotypes are presented in Table 7, and no significant risk of developing CRC was identified according to the haplotype variants.

## 4. Discussion

Interleukins-17A and -17F are proinflammatory cytokines produced primarily by Th-17 cells. They exert their effects on the cells of the immune system after binding to the IL-17RA and IL-17RC receptors, with the affinity of IL-17F for IL-17RA being 100 to 1000 times lower than that for IL-17A, while the affinity of the two cytokines for IL-17RC is similar [6]. In general, the proinflammatory effects of IL-17F, expressed by more cell types in the body, including epithelial cells, are weaker than those of IL-17A. The latter has been associated with the promotion of neoplastic transformation due to its ability to induce the expression of inflammatory cytokines and chemokines such as IL-1, IL-6, TNF, and GC-SF [24,25]. Moreover, in mouse experimental models, IL-17A overproduction has been shown to stimulate angiogenesis and metastasis [26]. In support of its pro-tumor effects, other studies have shown an association of increased Th-17 cell infiltration in tumor tissue with worse prognosis in lung cancer [27], CRC [13], hepatocellular carcinoma [28] and breast cancer [29]. In contrast, IL-17F overexpression appears to inhibit tumor progression in experiments with human and mouse models of colorectal and hepatocellular carcinoma [15,30].

Considering the above-discussed role of IL-17A and IL-17F in the pathogenesis of tumors of various origins, we aimed to investigate the effect of functional polymorphisms in the genes encoding these cytokines (*IL17A*-197A/G SNP, rs2275913 and *IL17F* 7488 T/C, rs763780), on the risk, progression and prognosis for CRC in patients from the Bulgarian population. Currently, these genetic variations are the subject of intensive research on their frequency in different populations, as well as their association with susceptibility to tumors. The variant A-allele with respect to *IL17A*-197A/G promoter SNP has been reported to affect gene transcription. This has been observed in vitro by stimulated T-cells from healthy individuals and the A/A- and A/G-genotypes secreted significantly higher levels of IL-17 than the G/G-genotype [31]. In the study by Liu et al. these observations are explained by the higher affinity of the A-allele for NFAT (nuclear factor of activated T-cells) transcription factor, obviously an important regulator of *IL17A* gene expression [13]. Therefore, the A/A-genotype could increase the susceptibility to CRC, leading to an inflammatory environment through elevated levels of IL-17A in the tumor microenvironment.

Our results showed that the genotype and allelic frequencies for *IL17A*-197A/G polymorphism differed significantly between the patient group and controls. The heterozygous carriers (A/G-genotype) presented with higher frequency in the control group, which showed a 2.39-fold statistically significant lower susceptibility to the development of CRC (*p* = 0.006). In addition, the A/G-genotype was associated with CRC at an earlier stage of progression (only 18.2% of patients in stage III or IV were heterozygous individuals, *p* = 0.024). Thus, our results indicate that heterozygosity for the rs2275913 polymorphism might have a protective role in the susceptibility to CRC in the Bulgarian population. Completely opposite data are reported by Nemati et al. According to the authors’ findings theA/G-genotype determines a significantly higher risk of CRC compared to A/A-carriers [17]. Such differences could be explained by population or ethnic characteristics, as well as the influence of other molecular factors on gene expression.

In a recent study on the effect of *IL17A* promoter polymorphism on the risk of CRC, the authors found that the A/A-genotype caused a significantly higher susceptibility to the disease in individuals of the Malaysian population, and no associations with clinical and histological features of patients [32]. Omrane et al. also found an increased risk of CRC for the A-allele but also reported that it was positively associated with a significant risk for late-stage disease [33]. We found a correlation between the A/A-genotype and the presence of distant metastases (only 7.9% of the cases of CRC without metastases were carriers of the A/A-genotype). In addition, this genotype correlated with an earlier age of diagnosis with CRC. Given the higher promoter activity of the *IL17A* gene in the presence of the variant allele, we could assume that the A/A-genotype contributes to the maintenance of inflammation and stimulation of angiogenesis in the growing tumor, which would lead to earlier CRC onset. In addition, our data showed that the survival of the studied patients was more favorable in the carriers of at least one G-allele (A/G and G/G-genotypes). Our data suggest that the A-allele of the studies SNP is not a risk factor for the susceptibility to CRC but affects the severity of the disease, and the heterozygous genotype provides a protective effect for the onset of the disease, given its significantly higher frequency among control individuals.

The single nucleotide variation 7488 T/C, located in exon 3 of the *IL17F* gene, has been studied in relation to susceptibility to various diseases such as ulcerative colitis, asthma, acute myeloid leukemia and various solid tumors, including CRC and gastric cancer [20,21,34,35]. Functional analses indicate that the replacement of the thymine nitrogen base by cytosine (T→C) in this polymorphism results in the substitution of histidine with an arginine residue at position 161 of the encoded polypeptide chain. This change is likely to inhibit the activity of the functional protein (IL-17F) and also inhibits the expression of the gene itself [34]. Data on the protective role of IL-17F in tumor progression due to inhibition of angiogenesis have been published, and its overproduction leads to a reduction in tumor formation in the colon (shown in mouse and human models) [15,30]. However, data on the role of rs763780 polymorphism in the pathogenesis of tumors in the gastrointestinal tract are conflicting. Some authors report an association of the T/C-genotype with an increased risk of lymph node metastasis as well as gastric cancer progression [18], while others report an increased risk of CRC with respect to the variant C-allele [17,36]. A study in the Tunisian population was conducted on the role of this polymorphism in the development of CRC and the authors found that the studied SNP is not a risk factor for predisposition to the disease, but genotypes containing at least one C-allele (T/C and C/C-) are presented with the lowest frequency in tumors with localization in the colon with high statistical reliability (*p* = 0.0001) [33]. In addition, the authors report an association of the same genotypes with tumor histology and architecture. Finally, the study deserves attention due to the hypothesis put forward by the authors, namely: *IL17F* 7488 T/C polymorphism is a genetic factor determining the increased risk of progression of a mucinous type of CRC with ulcerative architecture at a more advanced stage and higher risk of metastasis to surrounding lymph nodes.

In our study, we did not find a statistically significant difference in genotype and allelic frequencies between patients and controls, which gives us reason to suggest that rs763780does not play a significant role in susceptibility to CRC in the Bulgarian population. Our assumptions were supported by the lack of association of genotypes with the clinical and histological parameters of the studied patients. However, we found a trend of shorter survival of 19.9 months in heterozygous carriers compared to T/T-genotype carriers. Similar results have been published by Wu et al., who reported an increased risk of gastric cancer for the T/C-genotype [18]. Therefore, the hypothesis of the involvement of *IL17F* 7488 T/C polymorphism in the progression of CRC could not be ruled out.

As the *IL17A* and *IL17F* genes are located on the same chromosome, we aimed to determine if there was an association of haplotypes on both polymorphisms with the risk of CRC. Statistical processing of the data showed that haplotypes were not a risk factor in the pathogenesis of CRC. Similar results were reported by Nemati et al. [17].

## 5. Conclusions

Conclusively, our observations on the protective role of heterozygosity for the *IL17A*-197A/G SNP and the negative effects of the A-allele on CRC progression (increased metastatic recurrence and shorter survival) demonstrate that genetic factors are involved in colorectal cancer molecular pathology. Additionally, the role of the *IL17F SNP* in the overall survival of CRC cannot be excluded.

However, our results were obtained with a limited sample size, which is one major limitation of our statistical associations. Additional studies are needed to explore the association between the studied polymorphisms and the risk and progression of CRC among other ethnic populations.

## Figures and Tables

**Table 1:** PCR products, restriction fragments size and the respective genotypes for the *IL17A* and *IL17F* SNPs.

SNP	PCR Product Size (bp)	Restriction Enzyme	Restriction Product	
			Genotype	Restriction Products Size (bp)
*IL17A*-197 G/A	102	*XagI* (*EcoNI*)	AA	102
			AG	102, 68, 34
			GG	68, 34
*IL17F* 7488 T/C	143	*HinlII* (*NlaIII*)	CC	143
			CT	143, 80, 63
			TT	80, 63

**Table 2:** Demographic and clinical data of the studied CRC patients.

Parameter	Number (%)
Patients	*n* = 136
Gender	*n* = 136
Male	67 (49.3)
Female	69 (50.7)
Age at diagnosis (years)	*n* = 136
Range	44–88
Mean (±SD)	67.14 (±8.41)
Localization of primary tumor	*n* = 136
Colon	50 (36.8)
Rectum	65 (47.8)
Sigma	21 (15.4)
Local tumor invasion groups	*n* = 136
T 1-2 (early CRC)	37 (27.2)
T 3-4 (advanced CRC)	99 (72.8)
Grade of differentiation	*n* = 136
low	26 (19.1)
moderate	102 (75.0)
high	8 (5.9)
N staging	*n* = 136
N1-2	17 (12.5)
N0	119 (87.5)
M staging	*n* = 73
M1	18 (24.7)
M0	55 (75.3)
Survival status (total)	*n* = 73
alive	28 (38.4)
deceased	45 (61.6)

**Table 3:** Distribution of genotype and allelic frequencies of the *IL17A*-197 A/G SNP between CRC cases and controls.

*IL17A*-197G/A SNP	CRC patients		Controls		OR (95% CI), *p*-Value
	*Number*	Frequency	*Number*	Frequency	
	*n* = 104		*n* = 106		
Genotype					
AA	22	0.21	12	0.11	1.347 (0.591–3.072), *p* = 0.539
AG	33	0.32	58	0.55	0.418 (0.228–0.767), *p* = 0.006
GG	49	0.47	36	0.34	Ref. (1.0)
AA + AG vs GG (dominant model)	55	0.53	70	0.66	0.577 (0.331–1.007), *p* = 0.057
AG + GG vs AA (recessive model)	82	0.78	94	0.88	0.476 (0.222–1.021), *p* = 0.062
Allele					
A	77	0.37	82	0.39	0.932 (0.628–1.383), *p* = 0.763
G	131	0.63	130	0.61	Ref. (1.0)

**Table 4:** *IL17A*-197G/A SNP frequencies and OR in colon, rectal and sigma cancer cases and controls.

Genotype *IL17A*-197G/A	Controls *n* = 106	Colon *n* = 39	OR (95% CI)	*p*	Rectum *n* = 51	OR (95% CI)	*p*	Sigma *n* = 14	OR (95% CI)	*p*
*GG*	36	19	1.000 (reference)	-	25	1.000 (reference)	-	5	1.000 (reference)	-
*GA*	58	13	0.425 (0.187–0.963)	0.042	17	0.422 (0.201–0.888)	0.026	3	0.372 (0.084–1.653)	0.262
*AA*	12	7	1.105 (0.373–3.272)	1.000	9	1.080 (0.396–2.946)	1.000	6	3.600 (0.929–13.953)	0.074
Dominant *(GG* vs. *GA + AA)*	70	20	0.541 (0.257–1.141)	0.124	26	0.535 (0.271–1.056)	0.082	9	0.926 (0.289–2.967)	1.000
Recessive *(AA* vs. *GA + GG)*	94	32	0.584 (0.212–1.610)	0.404	42	0.596 (0.233–1.522)	0.319	8	0.170 (0.050–0.575)	0.007

**Table 5:** Genotype and allelic frequencies among CRC patients and control individuals according to the *IL17F* 7488 C/T polymorphism.

*IL-17F* 7488T/C	CRC Patients		Controls		OR (95% CI), *p*-Value
	Number	Frequency	Number	Frequency	
	*n* = 113		*n* = 116		
Genotype					
*TT*	107	0.95	110	0.95	1.0 (referent)
*TC*	6	0.05	6	0.05	1.028 (0.283–3.738), *p* = 0.963
*CC*	0	0	0	0	Na/N
Allele					
*T*	220	0.97	214	0.97	1.0 (referent)
*C*	6	0.06	6	0.03	0.973 (0.273–3.464), *p* = 0.962

**Table 6:** The IL17F 7488 T/C SNP was not associated with CRC stage and metastatic status of the patients.

	CRC Stage		*p* (χ^2^-Test)	N Stage		*p* (χ^2^-Test)	M Stage		*p* (χ^2^-Test)
*IL17F* 7488 T/C	T 1-2	T 3-4	0.353	N1-2	N0	0.278	M1	M0	0.589
TT	96.7%	92.6%		95.1%	88.2%		94.3%	88.2%	
CT	3.3%	7.4%		4.9%	11.8%		5.7%	11.8%	

**Table 7:** *IL17A* and *IL17F* haplotype distribution among CRC patients and controls.

IL-17A/IL-17F	CRC Patients		Controls		OR (95% CI), *p*-Value
	*n*	Frequency	*n*	Frequency	
	*n* = 81		*n* = 95		
Haplotypes					
G/T	48	0.593	57	0.597	1.0 (referent)
A/T	31	0.380	35	0.363	1.052 (0.541–2.043), *p* = 0.876
A/C	1	0.008	1	0.016	1.188 (0.031–44.855), *p* = 0.904
G/C	1	0.019	2	0.024	0.594 (0.021–8.737), *p* = 0.671

## Data Availability

Not applicable.
